# Understanding the P‐Cluster of Vanadium Nitrogenase: an EPR and XAS Study of the Holo vs. Apo Forms of the Enzyme

**DOI:** 10.1002/cbic.202400833

**Published:** 2024-12-02

**Authors:** Isis M. Wahl, Kushal Sengupta, Maurice van Gastel, Laure Decamps, Serena DeBeer

**Affiliations:** ^1^ Department of Inorganic Spectroscopy Max Planck Institute for Chemical Energy Conversion Stiftstrasse 34–36 Mülheim an der Ruhr 45470 Germany; ^2^ Department of Molecular Theory and Spectroscopy Max-Planck-Institut für Kohlenforschung Kaiser-Wilhem-Platz 1 Mülheim an der Ruhr 45470 Germany

**Keywords:** Nitrogenase, P-cluster, EPR, XAS, EXAFS, FeS cluster

## Abstract

The catalytic moiety of nitrogenases contains two complex metalloclusters: The M‐cluster (also called cofactor), where the catalytic reduction of substrates takes place, and the [Fe_8_S_7_] P‐cluster responsible for electron transfer. Due to discrepancies between crystallography and EPR spectroscopy, the exact structure of the P‐cluster in the VFe protein remains a topic of debate. Herein, we use an apo‐form of VFe (which retains the P‐cluster but lacks the FeVco) to study the VFe P‐cluster. SDS‐PAGE and NativePAGE showed a heterogeneous composition of the VFe and the apo‐VFe samples with the presence of α_1_β_2_δ_2_ and α_1_β_2_ complexes. The parallel mode EPR measurements of IDS oxidized MoFe, apo‐MoFe, and VFe samples reveal a signal at *g*=12 associated with the two‐electron oxidized state of the P‐cluster (P^2+^) for all three samples, albeit with different intensities. In contrast, no P^2+^ was observed for IDS oxidized apo‐VFe. Additionally, comparisons between apo‐MoFe, apo‐VFe and the model complex (NBu_4_)_2_[Fe_4_S_4_(SPh)_4_] via EXAFS measurements showed that apo‐VFe does not contain a fully formed [Fe_8_S_7_] P‐cluster, but rather is comprised of fragmented iron‐sulfur clusters. Our results point to a possible variation in the structure of the P‐cluster in the different forms of the nitrogenase.

## Graphical abstract

The present study brings a combination of biochemical techniques, electron paramagnetic resonance (EPR) spectroscopy and extended X‐ray absorption fine structure (EXAFS) data to help to understand the P‐cluster of nitrogenases, in particular the differences between the holo and apo‐forms of the nitrogenase VFe protein. It is demonstrated that apo‐VFe does not contain fully matured P‐clusters.

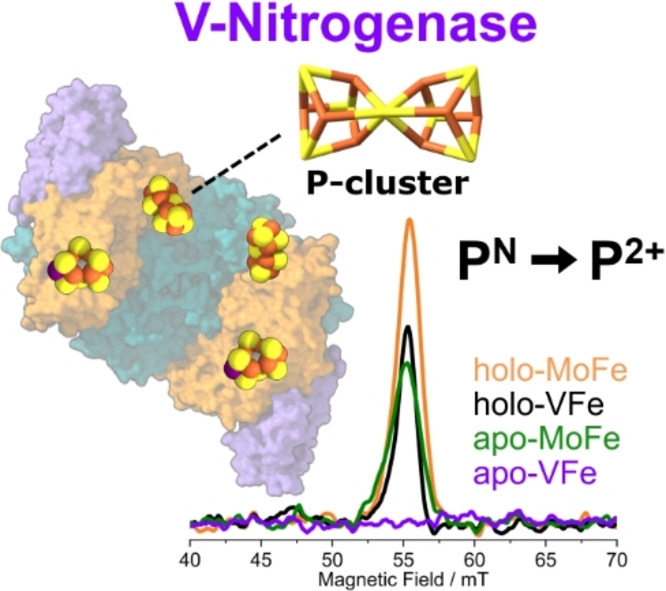

## 1 Introduction

**Figure 1:**
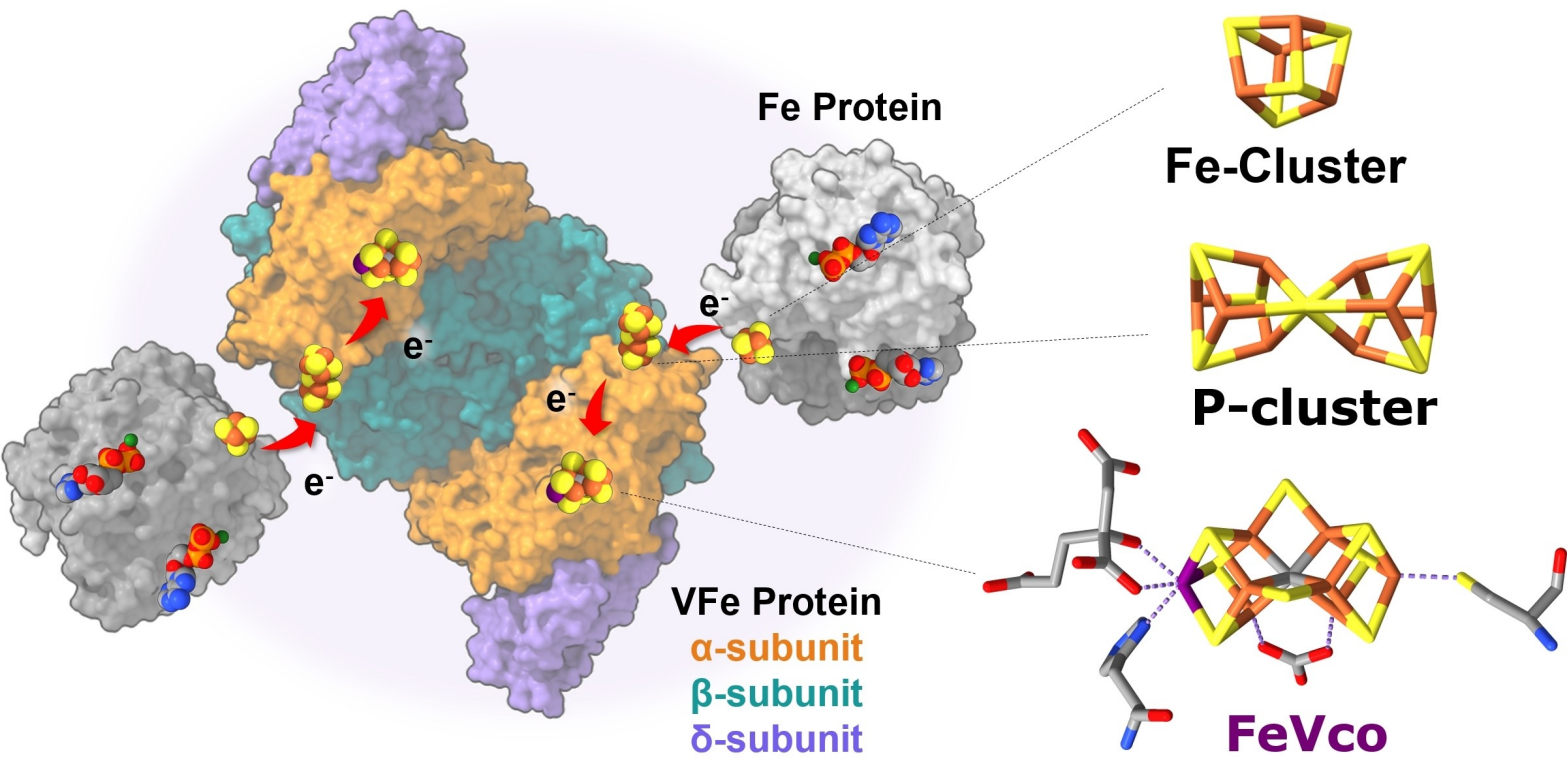
Rendering of the vanadium nitrogenase system, including the structures of the three metallocofactors (right). Iron is colored in orange, sulfur in yellow, carbon in gray, vanadium in purple, oxygen in red, and nitrogen in blue. The figures were generated using PDB IDs 5N6Y and 6Q93.

Nitrogenases are enzymes that catalyze the ATP‐dependent reduction of dinitrogen to ammonia, making nitrogen available for living organisms. These enzymes are only produced by a few identified bacteria, archaea, and one recently identified alga, which can use N_2_ as a nitrogen source.[^1^, ^2^] *Azotobacter vinelandii* is a widely used model organism because of its ability to produce all three known forms of nitrogenase isozymes: the Mo‐dependent, the V‐dependent, and the Fe‐only nitrogenases (named according to the composition of the active site/M‐cluster). These isozymes are two‐component protein complexes consisting of the homodimeric reductase iron protein, FeP, in which a [Fe_4_S_4_] cluster bridges the two subunits, and the catalytic protein, MFe, where M=Mo, V, or Fe, depending on the isozyme.[^3^, ^4^] The catalytic protein contains two complex metalloclusters: the [Fe_8_S_7_] P‐cluster responsible for electron transfer, and the M‐cluster or cofactor (FeMco) responsible for catalysis, which consists of [MoFe_7_S_9_C‐(*R*)‐homocitrate] for MoFe protein,^[5]^ [VFe_7_S_8_C(CO_3_)^2−^)(*R*)‐homocitrate] for VFe protein (Figure 1),[^6^, ^7^] or [Fe_8_S_9_C‐(*R*)‐homocitrate] for FeFe protein.[^8^, ^9^] The VFe protein is an α_2_β_2_δ_2_ hexamer encoded by the *vnfD*, *vnfK*, and *vnfG* genes.[^10^, ^11^]

Most of the attention and research on nitrogenases has historically been directed to the cofactor as it is the active site of this enzyme. However, it is important to acknowledge another important player in the functioning of nitrogenase: the P‐cluster. The P‐cluster has the fundamental function of promoting the electron transfer from the [Fe_4_S_4_] cluster of the FeP to the FeMco cluster. Crystallographic studies on MoFe from *A. vinelandii* showed that the P‐cluster is located at the interface between the α‐ and β‐subunits ~10 Å beneath the protein surface and ~14 Å away from the FeMo‐cofactor (FeMoco),[^12^, ^13^, ^14^] and that these distances are conserved in VFe^[7]^ and FeFe^[8]^ proteins. In the resting state, the P‐cluster in its fully reduced form (P^N^) has a backbone of two [Fe_4_S_3_] cubanes bridged by one central μ_6_‐sulfide. In MoFe, P‐cluster maturation occurs before FeMoco insertion. Two [Fe_4_S_4_] are fused through the sequential action of the NafH, NifW, NifZ, and NifH proteins.[^13^, ^14^, ^15^, ^16^] While NafH, NifW, and NifZ are accessory proteins, full maturation of the P‐cluster can occur only with NifH (the reductase component) or with a strong reductant such as Ti(III)citrate.^[17]^

Electron paramagnetic resonance (EPR) studies on MoFe revealed that the P‐cluster in its resting P^N^ form is EPR silent with an *S*=0 ground state.[^18^, ^19^] The EPR spectrum of the resting state of MoFe, *i. e*. reduced with reductants like dithionite (DT), exhibits only a rhombic *S*=3/2 signal, attributed to the FeMoco and no signal corresponding to the P^N^. One‐electron oxidation of P^N^ gives the mechanistically relevant P^1+^ state, exhibiting both *S*=1/2 (*g*=[2.06, 1.95, 1.82]) and *S*=5/2 (*g*_eff_=[6.67, 5.30, unknown]) signals.^[20]^ The P^2+^ state is generated by further oxidation and has an integer spin, likely *S*=4, presenting a specific signal at *g*=12 in the parallel mode.[^18^, ^21^] Although crystallographic studies of the MoFe and VFe proteins suggest that the structure of the P‐cluster is identical in both proteins, spectroscopic analyses have revealed notable differences.[^20^, ^22^]

In the DT‐reduced state, three distinct EPR signals have been reported for the VFe protein, corresponding to *S*=3/2, *S*=1/2 and *S*=5/2 spin systems. Generally, the *S*=3/2 signal has been attributed in the literature to the FeV‐cofactor (FeVco), while the *S*=1/2 signal has been suggested to arise from the reduced P‐cluster and the *S*=5/2 signal from the oxidized P‐cluster.[^23^, ^24^, ^25^, ^26^] Much of the previous spectroscopy and computational studies have thus utilized an *S*=3/2 ground state for interpretation of resting FeVco.[^20^, ^26^] However, more recent work by Yang *et al*. concluded that the FeVco in the resting state is likely diamagnetic (*S*=0) or possesses a non‐Kramer's integer spin (*S*=1, 2,…), analogous to the FeFe‐cofactor of Fe‐nitrogenase.^[24]^ Based on this, the authors suggested that the observed EPR active signals may arise either from a [Fe_4_S_4_]‐like cluster (*S*=1/2) or a catalytically inactive form of the FeVco (*S*=3/2).

The *S*=1/2 signal is one of the most unique and perplexing features of the VFe protein. This signal has primarily been associated with the P‐cluster, an assignment supported by studies that show that in the apo‐form of VFe (which retains the P‐cluster but lacks the FeVco), the *S*=3/2 signal is absent, while the *S*=1/2 signal persists.[^22^, ^24^] Hales *et al*. were the first to comment on the similarities between the *S*=1/2 present on the holo‐VFe protein and the *S*=1/2 signal present in the nitrogenase reductase (FeP from either VFe or MoFe) originating from the [Fe_4_S_4_] cluster.^[27]^ They observed that not only are the *g*‐values similar for both proteins, but also the effective spin concentration of this signal was low (approximately 0.4 spins per protein), which was attributed to the relative abundance of the *S*=1/2 and *S*=3/2 states.^[27]^ Further research by Hales and Blanchard led to the isolation of two forms of VFe protein from *A. vinelandii*, designated as “Av1′_A_” (α_1_β_2_) and “Av1′_B_” (α_2_β_2_). While both forms exhibited similar *S*=1/2 signals, with *g*‐values of 2.05 and 1.94, Av1′_A_ exhibited an additional signal with *g*=2.03, 1.93, and 1.89, which was attributed to a single [Fe_4_S_4_]^+^‐type cluster bound to the β‐subunit that was not coupled to an α‐subunit.^[28]^ This work demonstrated a critical aspect of the isolated VFe protein, its sample heterogeneity. The heterogeneity of VFe samples poses a significant challenge for spectroscopy, as discussed in a recent nuclear resonance vibrational spectroscopy study.^[29]^ Unlike the MoFe protein, which displays a well‐defined and quantifiable *S*=3/2 EPR signal, reports on the VFe protein have shown variability in signal intensity and broadening.[^20^, ^23^, ^25^, ^27^, ^30^] More recently, Yang *et al*. presented densitometry data that highlighted this heterogeneity in VFe samples.^[24]^ Their data showed that the β/α ratio on their VFe^Str^ samples was 1.1, while for the VFe^StrΔ*nifB*^ this ratio increased to 1.4. Yang and co‐workers suggested that this heterogeneity arises from variations in the α‐ and β‐subunit composition of the isolated VFe protein, which may contain both α_1_β_2_ and α_2_β_2_ species, as well as “spare” monomeric β‐subunits. Furthermore, the authors observed approximately ~0.3 spins per VFe^Str^ protein for the *S*=1/2 signal, consistent with previous reports,[^23^, ^31^, ^32^] indicating that this signal arises from a minor component of the sample. In a separate study, Hu *et al*. utilized a combination of EPR, X‐ray absorption spectroscopy (XAS), and activity assays to study P‐cluster variants.^[33]^ They came to the conclusion that both apo‐ (and by inference holo‐) VFe have P‐clusters comprised of two [Fe_4_S_4_]‐like fragments, and argued, in contrast to generally accepted views, that fully formed P‐clusters are not required for nitrogenase activity.

In order to understand the mechanism of how vanadium nitrogenase functions, it is crucial to gain a clear understanding of the resting state of the FeVco. However, as highlighted by ongoing debates in the literature, this area remains contentious, one particular reason being the discrepancies in the understanding of the VFe P‐cluster. In this work, we have sought to address these questions by conducting a parallel investigation that combines gel electrophoresis, EPR spectroscopy, and X‐ray spectroscopy on apo‐VFe and holo‐VFe and, in specific cases, on their MoFe counterparts under similar conditions. Herein, we demonstrate that VFe and MoFe in fact have very similar P‐clusters in the holo form of the enzymes, with spectroscopic differences arising from a fraction of immature P‐clusters. In contrast, we demonstrate that apo‐VFe does not seem to contain mature P‐clusters. The implications of these results for our understanding of P‐cluster maturation and for spectroscopic studies of VFe are discussed.

## Experimental

### Cell Culture

The *A. vinelandii* strains DJ2102, DJ2115, DJ2253, and DJ2256 used in the present study were generously provided by the group of Prof. Dennis Dean (Biochemistry Department of Virginia Polytechnic Institute and State University, USA). The linker‐ and Strep‐tag‐encoding sequence (ASWSHPQFEK) was placed at the N‐terminus of either of NifD (MoFe protein α‐subunit) in the case of DJ2102 and DJ2115 strains, or of VnfK (VFe β‐subunit) in the case of DJ2253 and DJ2256. The details of the strain construction can be found in Prof. Dean and co‐workers’ publications.[^14^, ^24^, ^34^]

The composition of media and general conditions used for the growth of *A. vinelandii* cells in the present work were based on procedures previously described.[^24^, ^34^, ^35^] The cells were grown in a modified Burke's medium in the presence of Na_2_MoO_4_ (0.01 M), for strains DJ2102 and DJ2115, or Na_3_VO_4_ (0.01 M), for DJ2253 and DJ2256. As a nitrogen source, NH_4_Cl was used in a final concentration of 10.5 mM in the precultures for all strains, and 4.5 mM in the main cultures, only for DJ2115 and DJ2256. In all cases, two 100 mL precultures were grown at 30 °C, under shaking at 180 rpm for 12 to 24 hours. These precultures were used to inoculate the main cultures, in which 10 mL of the precultures were added to each of the six 1 L main cultures. The main cultures were incubated at 30 °C under shaking at 180 rpm for 12 to 24 hours. For strains DJ2102 and DJ2253, the cells were harvested when reaching an optical density at 600 nm (OD_600_) between 1.6–2.5 by centrifugation for 25 min at 6500 rcf and stored frozen at −80 °C. For strains DJ2115 and DJ2256, the cells were allowed to grow until ammonium depletion in the medium, which happened, usually, when the cells reached an OD_600_ ~1.8–2.0. The ammonium content was tested using a semi‐quantitative Ammonia strips test (MACHEREY‐NAGEL^©^). The lack of NH_4_^+^ in the medium triggers the derepression of nitrogenase gene expression.[^35^, ^36^] For strains DJ2115 and DJ2256, cells were harvested 3 hours after derepression by centrifugation for 25 min at 6500 rcf and stored frozen at −80 °C.

### Purification of Strep‐Tagged Proteins

All protein isolation procedures were performed in anoxic conditions. The protein purification was performed via affinity chromatography using Strep‐Tactin® column material (IBA Lifesciences), following the manufacturer instructions^[37]^ and previously described literature procedures.^[34]^ The lysis of the cells was carried out using either BugBuster® Master Mix (Merck Chemicals^©^) or sonication. In the case where the BugBuster® Master Mix was used, before the cell lysis, the buffer was first degassed by stirring under N_2_ for 1 hour (in a Coy glovebox with a 98 % N_2_/2 % H_2_ atmosphere), then the cells were added, supplemented with 5 mM Na_2_S_2_O_4_ and allowed to stir for 1 hour. In the cases where sonication was used, the frozen cell pellets were added in deoxygenated 20 mM Tris/HCl, 200 mM NaCl buffer (pH=7.4), supplemented with 5 mM Na_2_S_2_O_4_ and one tablet of cOmplete™, EDTA‐free Protease Inhibitor Cocktail. The sonication was done in 5 cycles of 5 min with 50 % power in a Bandelin Electronic™ Sonopuls™ HD 2070 Homogenisator.

After the cell lysis process, the cells were transferred to air‐tight tubes and centrifuged for 1 hour at 4 °C and 45000 rpm (Beckman Coulter^TM^ Optima LE‐80 K, 70‐Ti rotor). After centrifugation, the soluble fraction was separated from the pellet and filtered using a 0.45 μm filter, then loaded onto a 10‐mL Strep‐Tactin® column, pre‐equilibrated with 20 mM Tris/HCl, 200 mM NaCl buffer (pH=7.4). After washing the column with 10 column volumes (CV) of this buffer, the protein sample was eluted with 3*CV 20 mM Tris/HCl, 200 mM NaCl, 3 mM desthiobiotin buffer (pH=7.4). The collected eluted protein solution was then concentrated using a 100 kDa MWCO centrifugal filter unit (Millipore). Protein concentrations were determined by Lowry assay^[38]^ and the protein purity was assessed via SDS‐PAGE with Coomassie staining.

### SDS‐Polyacrylamide Gel Electrophoresis Analysis

The protein samples were analyzed via SDS‐PAGE using Novex™ WedgeWell™ 4 to 20 %, Tris‐Glycine, 1.0 mm, mini protein gel from Invitrogen (Thermo Fisher Scientific). The samples containing between 2 and 4 μg of protein were mixed with Novex Tris‐Glycine SDS Sample Buffer and NuPAGE™ Sample Reducing Agent, following the manufacture procedures. In all cases, samples were heated for 5 min at 95 °C and loaded on the gel. The electrophoresis was performed using Novex Tris‐Glycine SDS Running Buffer (Invitrogen™) and, as a protein standard, 5 μL of SeeBlue™ Plus2 Pre‐stained Protein Standard was used. Gels were run for 2 h at 100 V. After the run, the gels were shortly washed with distilled water and then incubated in PageBlue Protein Staining solution for 1 hour under gentle shaking. After staining, the gel was rinsed and destained overnight in distilled water under gentle shaking. The destained gels were scanned using the BioRad Molecular Imager® Gel Doc™ XR+ System and the densitometry of the bands was analysed using the BioRad Image Lab 6.1 Software.

### Native‐Polyacrylamide Gel Electrophoresis Analysis

The protein samples were analyzed via NativePAGE under anaerobic conditions following published procedures.^[23]^ All buffers used in the electrophoresis were previously degassed and all procedures were performed in a Coy glovebox with a 98 % N_2_/2 % H_2_ atmosphere. The electrophoresis was performed using NativePAGE™ 4 to 16 %, Bis‐Tris, 1.0 mm, Mini Protein Gels from Invitrogen (Thermo Fisher Scientific). The precast gels were run for 1 h at 100 V without a sample in anoxic running buffer, to ensure no oxygen was present. To perform the electrophoresis NativePAGE™ Running Buffer, NativePAGE™ Cathode Buffer Additive, and NativePAGE™ Sample Buffer were used following the manufacturer instructions. Samples loaded contained between 2 and 4 μg of protein per well. NativeMark™ Unstained Protein Standard was used as the standard. The gels were run for 90 min at 150 V. After the run, the gels were shortly washed with distilled water and then incubated in PageBlue Protein Staining solution for 1 hour under gentle shaking. After staining, the gel was rinsed and destained overnight in distilled water under gentle shaking. The destained gels were scanned using the BioRad Molecular Imager® Gel Doc™ XR+ System and the densitometry of the bands was analysed using the BioRad Image Lab 6.1 Software.

### EPR Spectroscopy

All EPR samples were prepared in an anoxic atmosphere (in a Coy glovebox with a 98 % N_2_/2 % H_2_ atmosphere), 20 mM Tris/HCl, 200 mM NaCl buffer (pH=7.4), and at a concentration of 140 μM. Reduced samples were prepared with 5 mM of sodium dithionite (Na_2_S_2_O_4_). The indigo disulfonate (IDS)‐oxidized samples were prepared with a 10‐fold excess (~1.4 mM) of IDS. Thionine‐oxidized samples were prepared using adding the corresponding equivalent of thionine (0.5 to 3). All samples were incubated with the reducing or oxidant agent for at least 30 min. Oxidized samples were desalted using a 2 mL Dowex® 1×8 (100‐200 mesh) ion exchange resin column equilibrated in 20 mM Tris/HCl and 200 mM NaCl buffer, pH=7.4, and re‐concentrated using a 100 kDa MWCO centrifugal filter unit (Millipore) and then flash‐frozen in liquid nitrogen. All perpendicular‐mode and parallel‐mode EPR spectra were recorded using a Bruker X‐band ELEXSYS E500spectrometer equipped with a Bruker dual‐mode cavity (ER4116DM) and an Oxford Instruments ESR 900 liquid helium continuous‐flow cryostat. Perpendicular mode measurements were performed at 2 or 5 mW microwave power, at 0.746 mT/100 kHz modulation, and temperatures between 4 K and 30 K. Parallel mode measurements were performed at 200 mW microwave power, at 0.746 mT/100 kHz modulation and 14 K.

### X‐Ray Absorption Spectroscopy Measurements

Apo‐VFe data was collected at the I20‐scanning beamline, Diamond Light Source (DLS; Oxfordshire, UK), and apo‐MoFe data was collected at the P64 beamline, Deutsches Elektronen‐Synchrotron (DESY, Hamburg, Germany). The (NBu_4_)_2_[Fe_4_S_4_(SPh)_4_] complex was prepared as described in Averill *et al*.^[39]^ and measured at the 7–3 beamline, Stanford Synchrotron Radiation Lightsource (SSRL; Menlo Park, US).

The Fe K‐edge of apo‐VFe PFY‐XAS was collected at Diamond Light Source (DLS; Oxfordshire, UK) (3 GeV, 300 mA) at I20‐scanning,[^40^, ^41^] equipped with a four‐bounce Si(111) monochromator and rhodium‐coated harmonic rejection mirrors. This configuration provided a flux of ∼1×10^12^ photons/s (when unattenuated) at the sample position. The X‐ray beam approximated an unslitted, focused beam spot size of 0.3×0.4 mm^2^ (v×h; full width at half‐maximum (FWHM)). The Fe K‐edge of apo‐MoFe PFY‐XAS was collected at Deutsches Elektronen‐Synchrotron DESY (PETRA III, Germany) (6.083 GeV, 100 mA) at P64, equipped with a Si(111) monochromator providing a flux of ∼1×10^12^ photons/s (when unattenuated) at the sample position. The X‐ray beam had a focused beam spot size of 2×1 mm^2^ (v × h). The protein samples for XAS were prepared in the same buffer conditions as EPR with the addition of 5 mM DT. The concentration for the samples were about 0.5 mM. Each solution was transferred into a Kapton‐windowed Delrin cell and frozen in liquid nitrogen. During measurement, the sample cell was suspended in a top loading exchange gas pulse tube He cryostat, maintained at 10 K to minimize photodamage. Scans swept the incident energy from 6980 to 8214 eV, and the incident energy was calibrated by simultaneous measurement of a Fe foil, with the first inflection point of the Fe foil set to 7111.2 eV. Three ionization chambers – one before the sample (I0), one after the sample (It), and one after a reference foil (Iref) – were positioned along the beam path, and fluorescence data from the sample was recorded by a 64‐element monolithic Ge detector at I20‐scanning and by a 4‐element Si drift detector at P64. For the fluorescence detector, the readout was performed by an Xspress4 digital pulse processor. Damage assessment was performed prior to the collection of the full spectra via a series of short, low‐resolution scans at the near‐edge region. Flux was attenuated in order to ensure that each sample spot could survive a full EXAFS scan, and full spectra were collected at several fresh sample spots.

XAS data on the solid model complex were recorded in transmission mode at the Stanford Synchrotron Radiation Lightsource (SSRL) on beamline 7–3, under ring conditions of 3 GeV and 100 mA. A Si(220) double‐crystal monochromator was used for energy selection and detuned 50 % for harmonic rejection. Calibration of the incident beam energy was performed by assigning the first inflection point of the Fe foil spectrum to 7111.2 eV. The solid samples were prepared as a dilution in boron nitride and sealed with 38 μm Kapton tape windows. Measurements were made at 10 K in continuous flow liquid helium cryostat.

### X‐Ray Absorption Spectroscopy Data Processing

The Demeter software package was utilized for all data processing.^[42]^ Using the Athena module, the data were truncated to k=14 Å^−1^. Background subtraction and normalization were performed using a linear regression before the edge region from 6980 to 7082 eV was used, and a cubic polynomial regression was used for the region after the edge from 7262 to 8107 eV. The data were splined (k range: 0 to 16 Å^−1^, R‐background: 1.0, k‐weight: 2). The EXAFS was k^3^‐weighted (to enhance the impact of high‐k data) and Fourier transformed (FT) using a Hanning‐windowed k range: 2–14 Å^−1^.

## 2 Results and Discussion

### 2.1 Subunit Composition

**Figure 2:**
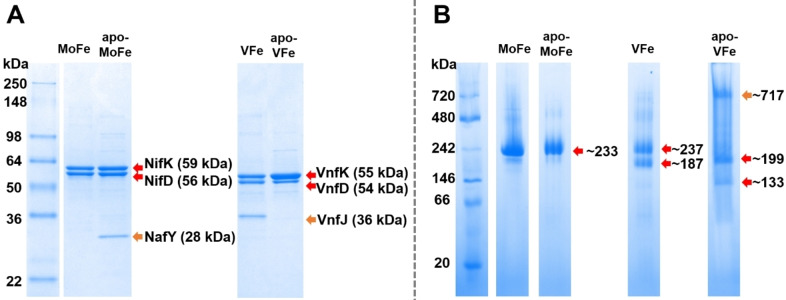
A) SDS‐PAGE Novex™ 4–20 %, Tris‐Glycine and B) NativePAGE™ 3–12 %, Bis‐Tris of MoFe, apo‐MoFe, VFe, and apo‐VFe. Red arrows indicate main species and orange arrows indicate additional bands.

The isolation of the nitrogenases of *A. vinelandii* via chromatography can follow several methods.[^7^, ^23^, ^34^, ^43^] To prioritize the integrity of potentially labile clusters, especially in the case of apo‐VFe, one‐step affinity purification of MoFe and VFe, tagged with a short Strep‐Tag sequence was performed (see Materials and Methods).[^34^, ^37^] After purification, the samples were analyzed via SDS‐PAGE (Figure 2A and Figure S1‐4), allowing identification and quantification of the subunits of the complexes, and NativePAGE (Figure 2B), allowing identification and quantification of the intact complexes. The well‐characterized MoFe and apo‐MoFe proteins were used as standards to ease the analysis of the VFe and apo‐VFe complexes.

The MoFe protein is characterized by an α_2_β_2_‐subunit composition with a molecular mass of 230 kDa. In contrast, the VFe protein has an α_2_β_2_δ_2_‐subunit configuration, resulting in a total molecular mass of approximately 240 kDa.^[28]^ However, in the case of apo‐VFe, the δ‐subunit (VnfG) is absent, resulting in an α_2_β_2_ composition for VFe^Str*ΔnifB*^ protein samples, with an expected molecular weight of around 214 kDa.[^7^, ^22^, ^24^] Following purification, isolated MoFe, VFe, apo‐MoFe, and apo‐VFe proteins were analyzed by SDS‐PAGE and NativePAGE (Figure 2). SDS‐PAGE enabled the resolution of the α and β‐subunits; for MoFe, these subunits weigh approximately 56 kDa and 59 kDa, respectively, whereas for VFe they are about 53 kDa and 54 kDa (Figure 2A). Additionally, VFe exhibited an extra band at 13 kDa, corresponding to the δ‐subunit, which is absent in apo‐VFe (see SI, Figure S5). Two additional bands could be observed: one in the apo‐MoFe sample at ~28 kDa, and one in the holo‐VFe sample at ~36 kDa (Figure 2A). Those bands were previously observed by Dean and colleagues following a Strep‐tag purification of these complexes, and attributed to NafY and VnfJ, respectively.[^14^, ^24^] According to the intensity of the band, each apo‐MoFe tetramer would be bound to one NafY protein, and each VFe heterohexamer would be bound to one VnfJ protein. Repetition of the VFe complex isolation procedure showed that the amount of VnfJ compared to VFe is variable. Further purification via size‐exclusion chromatography also revealed the presence of VFe complexes α_2_β_2_δ_2_ and α_1_β_2_δ_2_ not bound to VnfJ (see SI, Figure S5). Notably, while NafY has been shown to be involved in apo‐MoFe maturation, the role of VnfJ in VFe has not been elucidated.[^44^, ^45^]

**Figure 3:**
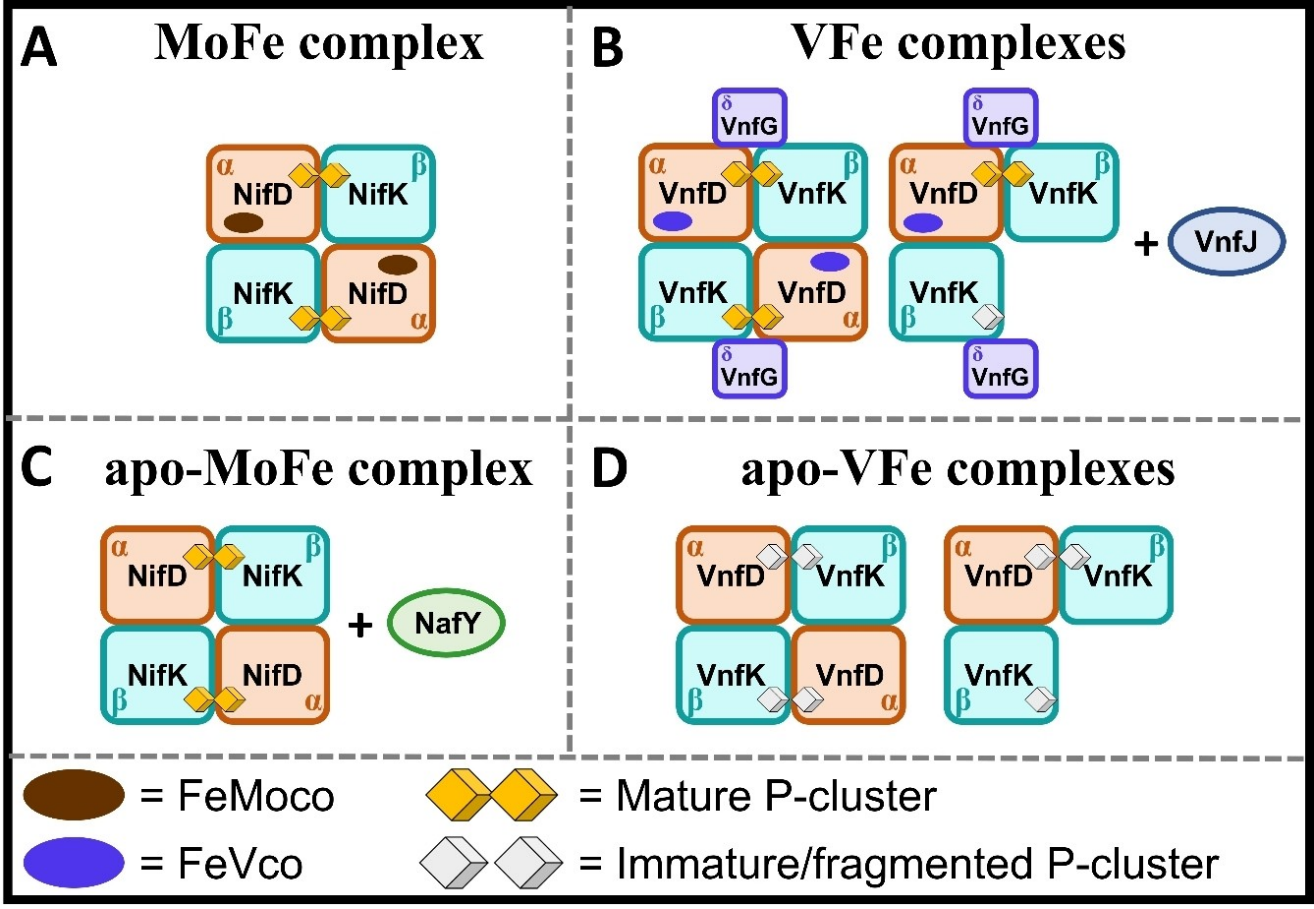
Schematics of the proposed clusters observed on the SDS‐PAGE and NativePAGE. (A) MoFe complex with α_2_β_2_‐subunit configuration. (B) VFe with α_2_β_2_δ_2_ and α_1_β_2_δ_2_‐subunit configurations. The gels also suggested the presence of VnfJ bound to these complexes, thereby forming VnfJ‐α_2_β_2_δ_2_ and VnfJ‐α_1_β_2_δ_2_. (C) apo‐MoFe complex with α_2_β_2_‐subunit configuration. The gels also suggested the presence of NafY bound to this complex, thereby forming NafY‐α_2_β_2_. (D) apo‐VFe with α_2_β_2_ and α_1_β_2_‐subunit configurations.

The NativePAGE analysis of MoFe and apo‐MoFe showed a unique band at ~233 kDa, corresponding to the full α_2_β_2_ complex (Figure 2B, Figure 3A). The binding of NafY in the case of apo‐MoFe only slightly affects the migration of the band (Figure 3C). The VFe sample exhibits a ~237 kDa band, corresponding to the VnfJ‐α_2_β_2_δ_2_ composition, and an additional band around 187 kDa of equal intensity (Figure 2B). This would suggest the presence of a VnfJ‐α_1_β_2_δ_2_ complex (Figure 3B). Meanwhile, the apo‐VFe lane unexpectedly displays three bands: the first one, at 717 kDa, likely corresponds to an aggregate of either the β‐subunit or a complex, implying the low stability of the protein when the cofactor is missing. The second band, at 199 kDa, would correspond to α_2_β_2_, and the third, at 133 kDa, to α_1_β_2_ (Figure 3D). These results are consistent with previous observations for these two forms of VFe.[^22^, ^24^]

For MoFe and apo‐MoFe, the β‐ and α‐subunits are present in equivalent amounts, as confirmed by integrating the area of the bands in the SDS‐PAGE gel (Table 1). In contrast, both VFe and apo‐VFe exhibit different ratios of the α‐ and β‐subunits. In VFe, the discrepancy is less pronounced, with a ratio of 1α to 1.5β. However, the imbalance is significantly more pronounced in apo‐VFe, which displays an α to β ratio of about 1 : 4. As indicated by the NativePAGE, the ratio between the α_2_β_2_ and the α_1_β_2_ forms is 1.3 : 1, implying that the excess of β‐subunit observed in the SDS‐PAGE does not only originate from the α_1_β_2_ form of apo‐VFe. Therefore, this excess may be attributed to the spare β‐subunit that formed the aggregate (Table 2).This excess of β‐subunit on the SDS‐PAGE of apo‐VFe was also observed by Hu *et al*., but it was not quantified.^[33]^ In the study by Yang *et al*., the SDS‐PAGE showed a 1α to 1.1β for holo‐VFe and a 1α to 1.4β on apo‐VFe.^[24]^ Clearly this variation is smaller than the one observed in the present work, which suggests the heterogeneity to be a consequence of slight differences in cell growth or protein purification. This variation could be also attributed to the potentially unequal formation of VnfK (β‐subunit) and VnfD (α‐subunit), or a difference in stability between those two subunits. Furthermore, given that the Strep‐tag used for purification is fused to the VnfK protein, the Strep‐tag purification process may result in the isolation of unbound VnfK alongside the complexes. This could also account for the presence of excess VnfK observed in the samples. Meanwhile, this method does not allow the isolation of unbound VnfD.

**Table 1:** The subunit ratio on nitrogenase samples from the densitometry scan of the SDS‐PAGE shown in Figure 2A.

Sample	Expected subunit composition	Additional band	β/α
MoFe	α_2_β_2_	n/a	0.98
apo‐MoFe	α_2_β_2_	NafY	1.05
VFe	α_2_β_2_δ_2_	VnfJ	1.46
apo‐VFe	α_2_β_2_	n/a	4.12

**Table 2:** Subunits and complex species, as identified on the NativePAGE shown in Figure 2B.

Sample	Expected subunit composition	Expected MW	Actual subunit composition	Molecular weight (kDa)
MoFe	α_2_β_2_	230	α_2_β_2_	233
apo‐MoFe	NafY‐α_2_β_2_	258	NafY‐α_2_β_2_	233
VFe	VnfJ‐α_2_β_2_δ_2_	276	VnfJ‐α_2_β_2_δ_2_	237
			VnfJ‐α_1_β_2_δ_2_	187
apo‐VFe	α_2_β_2_	214	β‐subunit aggregate	717
			α_2_β_2_	199
			α_1_β_2_	133

The lack of δ‐subunit VnfG along with the VnfD and VnfK subunits in apo‐VFe is a possible explanation as to why separated subunits are observed.^[28]^ The role of the δ‐subunit remains elusive, as it has been suggested to be involved in the insertion of the cofactor during maturation, the binding of the reductase, or the channeling of the substrate to the active site. Interestingly, structural studies of the iron nitrogenase complex lacking the δ‐subunit using cryo‐electron microscopy could not resolve electron densities for a part of the α‐ and β‐subunits including the P‐cluster and its binding site, implying an influence of the δ‐subunit on this region.^[46]^ Consequently, the absence of VnfG has a strong influence on the structure of the complex and could potentially result in VnfDK adopting an unstable conformation.

### 2.2 EPR Spectroscopy

#### 2.2.1 Reduced State

**Figure 4:**
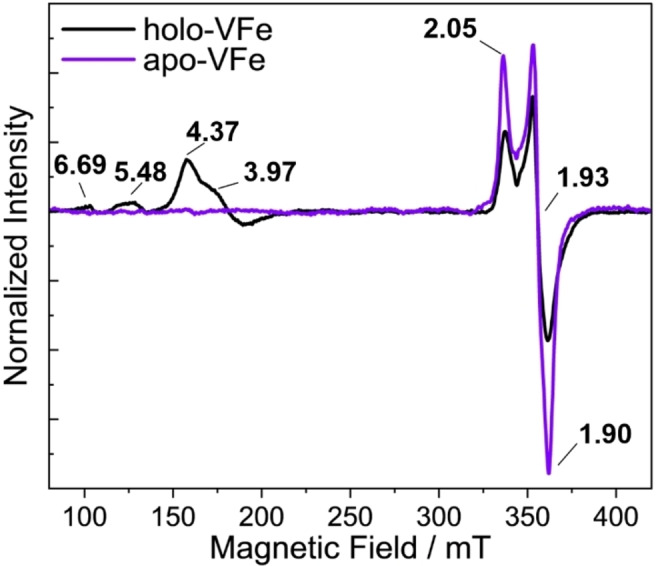
Perpendicular‐mode X‐band EPR of VFe (black) and apo‐VFe (purple) proteins. Both samples were at a concentration of 140 μM and were reduced with 5 mM DT. EPR conditions: temperature 14 K; microwave frequency 9.63 GHz; microwave power 5 mW; modulation amplitude 7.46 G. Each trace is the average of 5 scans.

The X‐band EPR spectra for both holo‐VFe and apo‐VFe in their DT‐reduced forms are shown in Figure 4. The EPR spectra of holo‐VFe could be divided into two regions: the low field region (*g*=5~3) and the high field region (*g*~2). In the low field region, multiple broad signals with *g*‐values of 5.48, 4.37, and 3.97 were observed, corresponding to a combination of different spin systems with *S*=3/2 and *S*=5/2. In the high field region, a slightly rhombic *S*=1/2 EPR signal is observed with *g*‐values of 2.05, 1.93, and 1.90.[^23^, ^24^, ^47^] This high field signal was also observed in the apo‐VFe sample, although the low field signals were absent. The *S*=1/2 signal in the apo‐VFe sample was approximately 65 % more intense, as determined by the integration of the areas of the absorption spectra of these signals. These results are consistent with previously published work on these proteins.[^23^, ^24^, ^47^] The *S*=1/2 signal has been associated with an increase in sample heterogeneity, specifically an excess of the β‐subunit relative to the α‐subunit in isolated samples of VFe. This deficit in the α‐subunit may have impeded P‐cluster maturation, giving rise to signals from immature P‐clusters or a FeS fragment. Recently, Van Stappen *et al*. employed EPR and X‐ray absorption spectroscopy to analyze two MoFe species (^*ΔHΔZ*^NifD_2_K_2_ and ^*ΔHΔZ*^NifD_2_K_2_W_2_), isolated from *A. vinelandii*, bearing immature P‐clusters.^[15]^ An *S*=1/2 signal was also detected in the EPR spectrum of both DT‐reduced ^*ΔHΔZ*^NifD_2_K_2_ and ^*ΔHΔZ*^NifD_2_K_2_W_2_ species, with *g*‐values of [2.05, 1.93, 1,90] and [2.04, 1.93], respectively, and was attributed to the presence of immature P‐clusters. With a combination of EPR and XAS measurements, they identified these immature clusters as two [Fe_4_S_4_]^2+/+^ clusters in close proximity.

To further characterize the clusters, temperature‐dependent EPR experiments were performed on apo‐VFe (see SI, Figure S6). In the high field region, the characteristic *S*=1/2 signal exhibited a maximal intensity at 20 K, with *g*‐values of 2.05, 1.95, 1.90, and a small shoulder at *g*=1.92. The shape and *g*‐values of this signal are similar to those reported by Van Stappen *et al*. for DT‐reduced ^*ΔHΔZ*^NifD_2_K_2_, as discussed in the previous section.^[15]^

#### 2.2.2 IDS Oxidation

The oxidant 5,5′‐indigodisulfonic acid sodium salt (IDS) has been extensively used to oxidize nitrogenases. Because of its relatively low redox potential (*E_0_* =−125 mV vs NHE, pH=7), IDS is not capable of oxidizing the FeMoco (M^N/OX^ *E_0_* =−42 mV vs NHE, pH=7.5);^48^ however, it effectively oxidizes the P‐cluster in MoFe and generates the P^2+^ state when used in excess.[^49^, ^50^] This distinctive property makes IDS an ideal oxidant for studies focusing on the P‐cluster. In the P^2+^ state, the P‐cluster acquires an integer spin state, *S*=4 or *S*=3 (as demonstrated in MoFe), which is identifiable in parallel‐mode EPR spectroscopy as a signal at *g*=12.[^21^, ^51^] This signal is specific to the P‐cluster in the P^2+^ state and is not observed in IDS‐oxidized MoFe carrying immature P‐clusters.^[52]^

**Figure 5:**
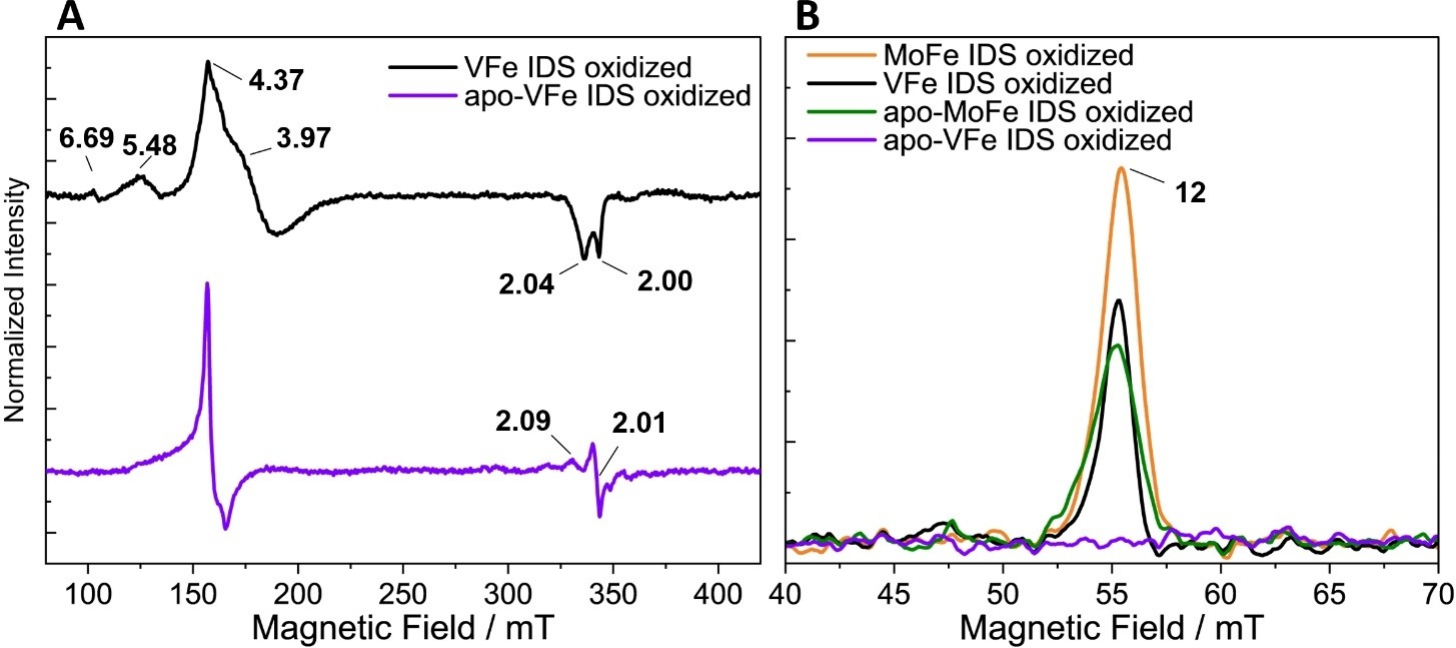
(A) X‐band perpendicular mode EPR of VFe (black) and apo‐VFe (purple). (B) X‐band parallel mode EPR of MoFe (orange), VFe (black), apo‐MoFe (green) and apo‐VFe (purple). All samples were at a concentration of 140 μM and oxidized by 10x excess of IDS. EPR conditions: temperature, 14 K; microwave frequency, 9.63 GHz (perpendicular‐mode) and 9.32 GHz (parallel‐mode); microwave power, 5 mW (perpendicular‐mode) and 200 mW (parallel‐mode); modulation amplitude, 7.46 G; Each trace is an average of 10 scans.

Based on the work of Minteer *et al*., where the P‐cluster in VFe was shown to have a similar redox potential to that of MoFe, IDS was employed in this work to investigate the oxidation of the P‐cluster in VFe.^[49]^ As shown in Figure 5A, upon IDS‐oxidation (in excess) of VFe, the low field region (*g*=6.69, 5.48, 4.37) in the perpendicular mode is essentially unaltered (relative to the data shown in Figure 4), while in the high field region, the *S*=1/2 (*g*=~2.0) signal is dramatically decreased. In contrast, the oxidation of apo‐VFe (Figure 5A) results in two signals with *g*=4.3 and *g*=2.0. For both VFe and apo‐VFe the re‐reduction of the oxidized samples (see SI, Figure S7) brings back the features observed in the intial reduced samples (Figure 4), showing that the oxidation process is reversible.

MoFe and apo‐MoFe were used as standards to evaluate the IDS‐oxidation in the parallel mode (Figure 5B). Both MoFe and apo‐MoFe exhibit a *g*=12 signal upon oxidation with excess IDS, as described in previous studies.^[53]^ Surprisingly, the intensity of the apo‐MoFe signal is lower than that of MoFe. The apo‐MoFe signal has been previously reported by Christiansen *et al*.; however, in that study, the authors did not discuss the relative intensity of the MoFe and apo‐MoFe signals.^[53]^ The crystal structure of apo‐MoFe suggests an almost identical P‐cluster site, while the αIII domain undergoes major conformational changes upon insertion of the FeMoco.^[54]^ The difference in intensity in apo‐MoFe might be explained by a slight distortion at the P‐cluster site due to the different conformation of this domain or by the binding of NafY, which was evidenced by our SDS‐PAGE and NativePAGE analyses (Section 3.1). Interestingly, the *g*=12 signal also appears less intense in apo‐MoFe than in holo‐MoFe in the study by Christiansen *et al*., however, the electrophoresis gel analysis of the proteins does not show any other proteins such as NafY, suggesting that the lower intensity of the *g*=12 signal is not due to the binding of this accessory protein.^[53]^

The *g*=12 signal could also be observed for IDS‐oxidized VFe (Figure 5B). While an early report from Tittsworth and Hales mentioned the presence of this signal, this is the first time that a spectrum has been published showing the *g*=12 signal in VFe. In contrast, Ribbe and co‐workers have previously published a parallel‐mode EPR spectrum of IDS‐oxidized VFe that shows no distinguishable signal.[^22^, ^23^] Based on that result, they suggested that VFe might possess a different P‐cluster than that found in MoFe. However, as discussed above, crystallographic data suggested that the P‐cluster in VFe is identical to that in MoFe; our result confirms the crystallographic observation. Notably, the EPR signal is less intense than in MoFe and is comparable to apo‐MoFe. One possible explanation for this discrepancy could be the well‐documented heterogeneity of VFe. Both our own gel electrophoresis results (Figure 2) and previous literature reports suggest an inhomogeneous distribution of α/β subunits. In our experiments, this ratio was as high as 1 : 1.5 (α : β), indicating the presence of an α_1_β_2_ complex, which was shown in the NativePAGE to be present in nearly equal amounts as the α_2_β_2_ complex. Consequently, this mixture of α_1_β_2_ and α_2_β_2_ complexes contains a lower quantity of fully formed P‐clusters. Our gel electrophoresis experiments also suggested that those complexes are bound to VnfJ, whose binding site is unknown. The effect of the binding of this protein to the complex on the spectroscopic properties of the enzyme is also unknown.

**Figure 6:**
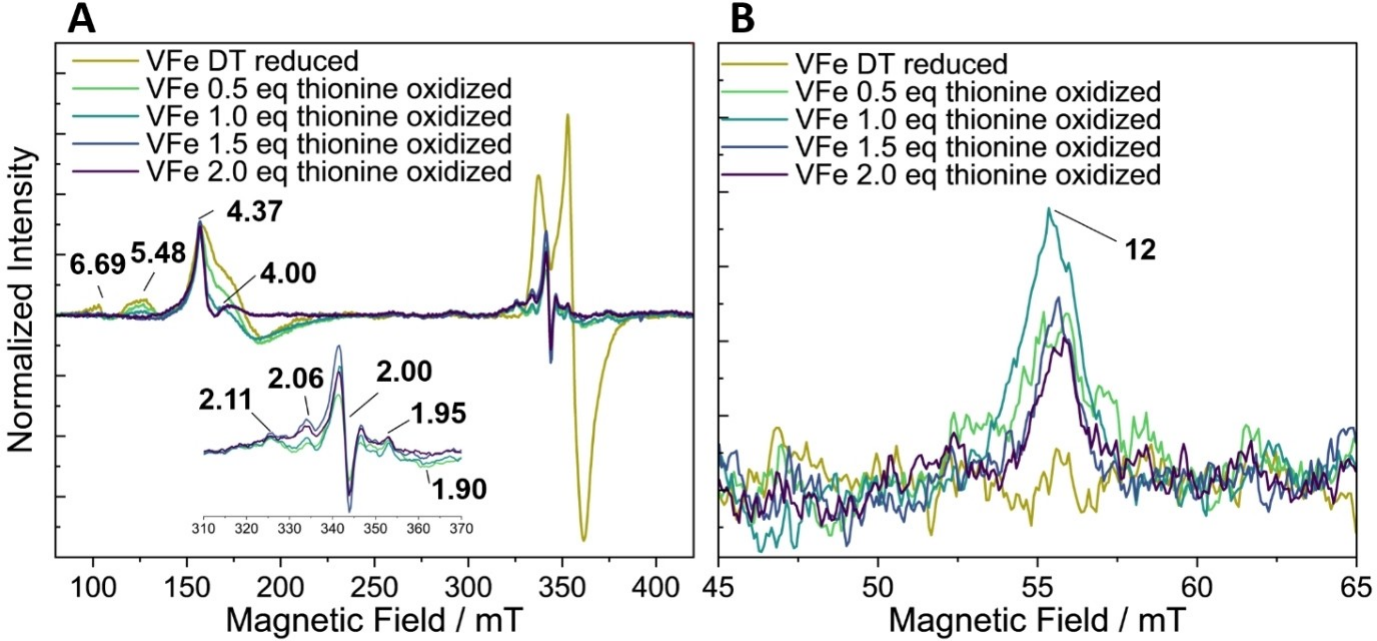
X‐band EPR spectra of VFe protein oxidized with thionine as indicated in the graph. EPR spectra measured in perpendicular‐mode (A) and parallel‐mode (B). EPR conditions: protein concentration 140 μM, temperature 14 K; microwave frequency, 9.63 GHz (perpendicular‐mode) and 9.32 GHz (parallel‐mode); microwave power, 5 mW (perpendicular‐mode) and 200 mW (parallel‐mode); modulation amplitude, 7.46 G; Each trace is the average of 10 scans.

Surprisingly, no *g*=12 signal could be observed in IDS‐oxidized apo‐VFe. This was unexpected, as our gel electrophoresis analyses showed the presence of α_1_β_2_ and α_2_β_2_ complexes, and suggested a structural or electronic difference of the P‐clusters in apo‐VFe compared to apo‐MoFe or VFe. In the case of apo‐VFe, no crystal structure has yet been published. P‐cluster maturation has been well studied in MoFe, using genetic knockouts to identify the role of accessory proteins and of the reductase in the fusion of two [Fe_4_S_4_] clusters to form the P‐cluster.[^14^, ^16^, ^52^, ^55^] In MoFe, P‐cluster maturation is completed before the insertion of the FeMoco. Meanwhile, P‐cluster maturation in VFe might happen differently. Previous studies and this one evidenced the lack of δ‐subunit in apo‐VFe, implying that FeVco, or its insertion, is required for the δ‐subunit to be bound.[^22^, ^24^] P‐cluster maturation in VFe might be linked to cofactor insertion, binding of the δ‐subunit, or an unknown step.

#### 2.2.3 Thionine Oxidation

**Figure 7:**
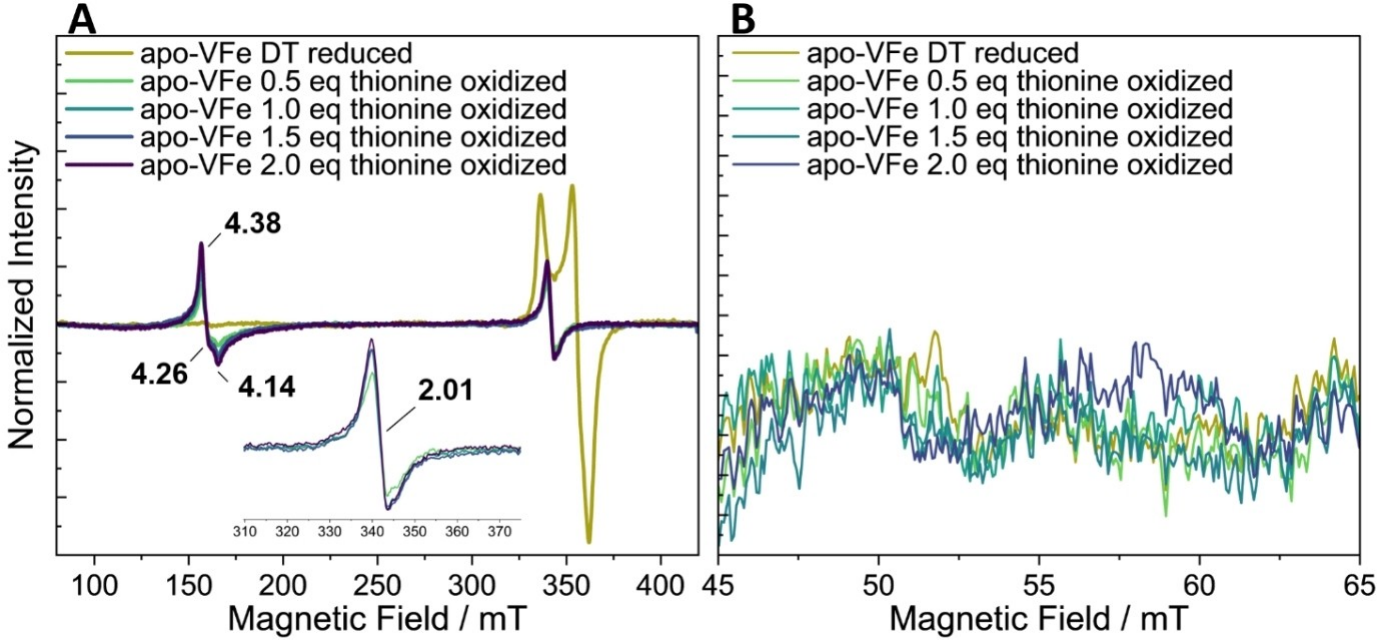
X‐band EPR spectra of apo‐VFe protein oxidized with thionine as indicated in the graph. EPR spectra measured in perpendicular‐mode (A) and parallel‐mode (B). EPR conditions: protein concentration 140 μM, temperature 14 K; microwave frequency, 9.63 GHz (perpendicular‐mode) and 9.32 GHz (parallel‐mode); microwave power, 5 mW (perpendicular‐mode) and 200 mW (parallel‐mode); modulation amplitude, 7.46 G; Each trace is the average of 10 scans.

Thionine has previously been used in nitrogenase research, in order to stepwise oxidize the P‐cluster, thereby gaining insights into the nature of P^+^, P^2+^, and P^3+^.[^25^, ^56^] Additionally, due to its relatively higher redox potential (~62 mV) compared to IDS, thionine has also been used to oxidize the resting cofactor to its 1e^−^ oxidized state.^[56]^ Thus in addition to the studies above using IDS, oxidation was also investigated utilizing thionine. During the stepwise oxidation of both VFe and apo‐VFe, significant changes were observed in the *g*=2 region, where a new signal appears in the EPR with the first addition of thionine (0.5 equivalent/protein) (Figures 6A and 7A). In the case of holo‐VFe, in addition to the modulations in the *g*=2 region, additional less intense signals, with *g* ~2.11, 2.06, 1.95, and 1.90, emerged in the high field region (Figure 6A, inset), which can be attributed to the formation of the P^+^ state of the P‐cluster, as previously observed in a similar state in MoFe.^[56]^ Conversely, in the low field region of the spectra, the features of the *S*=3/2 signal did not exhibit the same behavior. The signals at *g*~4 and 5.48 gradually decreased in intensity with increasing equivalents of thionine, reaching a minimum intensity at 2 equivalents of thionine/protein (Figure 6A). Interestingly, the intensity of the peak at *g*=4.37 remained unchanged throughout the entire titration, indicating either that the signal may be associated with a species that has a higher redox potential than thionine, or that the decrease in signal intensity is masked by the appearance of a more intense signal at the same *g*‐value with the addition of thionine. Notably, for apo‐VFe upon oxidation, there was an emergence of a signal at *g*=4.38, as also seen with IDS (Figure 7A). However, no P^+^‐signal could be observed upon oxidation of this sample. In parallel mode, the P^2+^ signal reaches a maximum at 1.0 eq of thionine/protein for holo‐VFe (Figure 6B). This stoichiometry is surprising, as we would expect that 2.0 eq would be necessary to oxidize 2 P‐clusters/protein from P^N^ to P^2+^. This suggests that VFe contains less than 2 mature P‐clusters, consistent with the results of the IDS oxidation discussed before. The importance of investigating the different stages of oxidation for both the cofactor and the P‐cluster can be recognized, however, the inherent inhomogeneity presents challenges in making definitive conclusions about these oxidation stages. For apo‐VFe no signal was observed in the parallel mode (Figure 7A), consistent with the oxidation with IDS.

### 2.3 EXAFS Analysis

**Figure 8:**
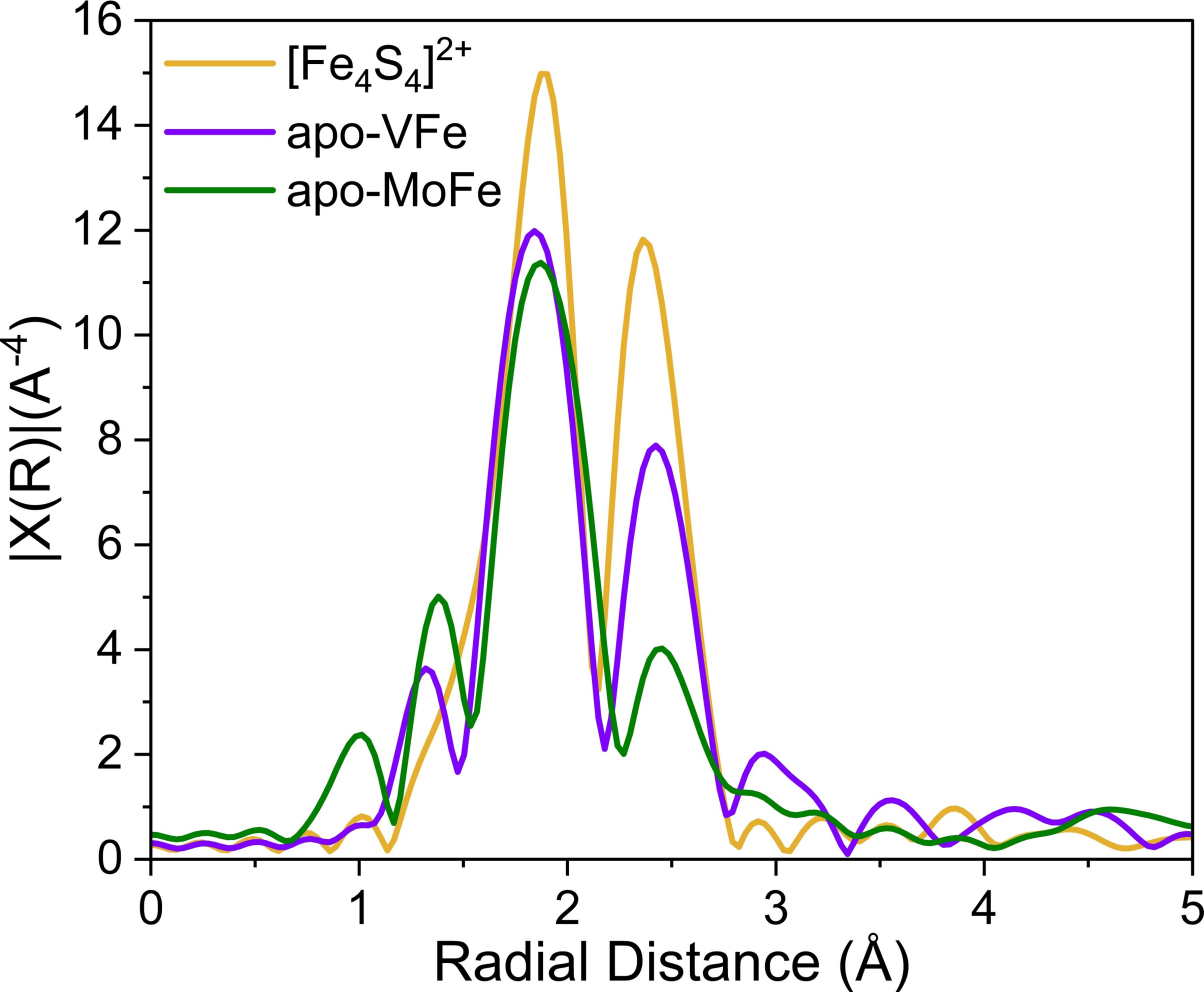
X‐ray absorption spectra of (NBu_4_)_2_[Fe_4_S_4_(SPh)_4_] (yellow), apo‐VFe (purple), apo‐MoFe (green). Non‐phase shifted EXAFS Fourier transform over a *k*‐range of 2 to 14 Å^−1^.

The irregular nature of the P‐cluster in the apo‐VFe sample, along with EPR, is also evident in the Fourier transform (FT) EXAFS data collected on the apo‐VFe protein. Figure 8 presents the FT EXAFS data for both apo‐MoFe and apo‐VFe, alongside a reference (NBu_4_)_2_[Fe_4_S_4_(SPh)_4_] molecular cluster for comparison. In the apo‐MoFe sample, the data clearly indicate the presence of an intact and mature P‐cluster (Figure 8, green), consistent with the previous report by Musgrave *et al*. EXAFS studies on both the reduced and oxidized P‐clusters of MoFe protein have revealed the FT data is dominated by a strong Fe−S 1^st^ shell scattering peak.^[57]^ This feature is complemented by a weaker Fe−Fe peak. For the reduced P‐cluster, this feature appears around 2.5 Å, which arises from two separate Fe−Fe vectors: a shorter one at approximately 2.5 Å and a longer one near 2.75 Å. As these two Fe−Fe vectors have EXAFS waves that are nearly out of phase, this results in diminished Fe−Fe amplitude in the FT. This is in contrast to isolated Fe_4_S_4_ clusters, where a single Fe−Fe vector at 2.75 Å (Figure 8, yellow) results in a pronounced second shell feature, which confirms that the P‐cluster of MoFe does not contain two symmetric Fe_4_S_4_ clusters, but rather a fused cluster which leads to the origin of the shorter Fe−Fe distance. FT EXAFS spectrum of apo‐VFe, containing a more reduced Fe−S cluster as observed in the XANES spectra (see SI, Figure S8), shows significant deviations from that of apo‐MoFe and more closely resembles the model Fe_4_S_4_ cluster with a stronger second shell Fe−Fe feature (Figure 8, purple). This provides further support for the presence of fragmented/immature P‐clusters in apo‐VFe.

## 3 Discussion

While the three nitrogenase isozymes can reduce N_2_ in NH_3_ and show strong structural similarities in their protein scaffold as well as their cofactors, they exhibit significant differences in their reactivities. Notably, V‐nitrogenase can reduce CO in hydrocarbons.^[58]^ The V‐nitrogenase catalytic protein contains two metal clusters: the P‐cluster and the M‐cluster (FeVco). While nitrogen binding and reduction occur at the M‐cluster, the P‐cluster plays a fundamental role in electron transfer, without which the reaction cannot proceed. Most studies on the P‐cluster have been performed using Mo nitrogenase, allowing the characterization of its structural and electronic properties as well as its maturation. Crystal structures are available for MoFe,^[59]^ apo‐MoFe,^[54]^ and VFe^[7]^ and show a near‐identical P‐cluster structure and location. Electrochemistry studies also showed similar redox potentials for the resting/singly oxidized P‐cluster redox couple (P^N/+^) in MoFe, apo‐MoFe, VFe, and apo‐VFe.^[49]^ Based on this similarity, one would expect that apo‐VFe would also bear similar P‐clusters, however, our current findings suggest otherwise. Discrepancies in the EPR spectra of both VFe and apo‐VFe have been previously noted in the literature.[^23^, ^24^, ^28^] While the *S*=3/2 signal has been previously associated with FeVco^[23]^ and more recently an inactive form of the cofactor,^[24]^ the origin of the *S*=1/2 remains a subject of ongoing investigation.[^23^, ^24^, ^33^] Our EPR analysis shows that the *S*=1/2 signal persists in the apo‐VFe sample, while the *S*=3/2 signal disappeared, in agreement with previously published works. Another important observation is that the *S*=1/2 signal, which has g values of *g*=[2.05, 1.95, 1.90] in both apo and holo‐VFe, is about 65 % more intense in apo‐VFe compared to holo‐VFe. This suggests that the species responsible for this signal is more abundant in apo‐VFe, indicating notable differences between the species present in apo‐ and holo‐VFe. The nature of the species producing the *S*=1/2 has been speculated in previous works.[^23^, ^24^, ^28^] These studies have linked this signal to sample heterogeneity, attributing it to minor components, likely FeS‐cluster fragments. These results are reminiscent of a previous report from our group by Van Stappen *et al*. which found similar *S*=1/2 signals for DT‐reduced immature P‐clusters in the MoFe protein produced in a Δ*nifH* Δ*nifZ* background.^[15]^ Another fundamental similarity was also observed in the EXAFS analysis of Van Stappen *et al*., where immature P‐cluster containing samples display a feature at ~2.4 Å in the FT, similar to the one observed on apo‐VFe at 2.5 Å and also for [Fe_4_S_4_] cubane clusters.[^13^, ^57^, ^60^]

Analysis of parallel‐mode EPR data allowed the two‐electron oxidized state P^2+^ to be probed. In this state, the P‐cluster has an integer spin (*S*=3 or 4), which is not visible in the perpendicular mode EPR. Our results show that both IDS‐oxidized MoFe and VFe exhibit a *g*=12 signal. While this signal was well described in the literature for MoFe, previous works from Lee et al.^[23]^ had reported that VFe did not exhibit any measurable signal. Our results are consistent with the observation of an identical P‐cluster in the high‐resolution crystal structures of VFe and MoFe[^7^, ^59^]. We also extended our parallel‐mode EPR studies to apo‐MoFe and apo‐VFe. For apo‐MoFe, we detected a signal that was less intense than the one displayed by MoFe, similar to previous reports^[53]^, however for apo‐VFe, no signal could be detected. A similar result was obtained when thionine was used as an oxidant. The stepwise oxidation of VFe led to the observation of a signal in the high field region that can be attributed to the one electron‐oxidized P^+^ state, which did not appear in apo‐VFe. These results suggest that no P^+^ or P^2+^ cluster is present on oxidized apo‐VFe.

Additionally, the fact that the P^2+^ intensity of the holo‐MoFe protein is almost double the intensity of holo‐VFe is consistent with the inhomogeneity of the VFe samples. In fact, such inhomogeneity was observed on the SDS‐PAGE and NativePAGE of both VFe and apo‐VFe. On the SDS‐PAGE, it is possible to see a clear heterogeneity in the composition of VFe and apo‐VFe, showing an α/β‐subunit proportion of 1 : 1.5 and 1 : 4 respectively. While on the NativePAGE, bands corresponding to at least 2 complexes could be identified in each, being a VnfJ‐α_2_β_2_δ_2_ and VnfJ‐α_1_β_2_δ_2_ for VFe and α_2_β_2_ and α_1_β_2_ for apo‐VFe. In apo‐VFe, the NativePAGE also suggested the presence of an aggregate of spare β‐subunits. The formation of a mature P‐cluster requires an equal amount of α‐ and β‐subunits. Hence, the 1 : 1.5 α/β ratio in VFe suggests that only 2/3 of the protein complex contains a mature P‐cluster, which would account for the lower intensity of the *g*=12 signal. However, the lower α/β ratio in apo‐VFe does not explain the complete lack of *g*=12 signal.

Our combined results of EPR and XAS analyses suggest that apo‐VFe does not contain a fully formed mature [Fe_8_S_7_] P‐cluster, but rather fragmented iron‐sulfur clusters. This implies that in VFe spectroscopic studies, apo‐VFe cannot be used as a standard to subtract the P‐cluster signal from a holo‐VFe sample to study the cofactor. Understanding the reactivity of nitrogenases requires the use of various advanced spectroscopic techniques such as EPR, Mössbauer, or XAS.^[20]^ Fe‐XAS comparing resting‐state and one electron‐reduced sample of MoFe revealed an Fe‐centered reduction as the first step of the MoFe cycle^[61]^. As extracted M‐cluster can be inserted in apo‐MoFe to reconstitute holo‐MoFe, apo‐MoFe has also been used as a scaffold for studying the nitrogenase reaction and the characteristics of the M‐cluster.^[62]^ This property has also been used to investigate the different reactivity of the cofactors, by inserting FeMoco or FeVco in apo‐MoFe.^[63]^ The use of apo‐MoFe for these studies reflects the cellular process of MoFe maturation, in which cofactor insertion is the last step and follows P‐cluster maturation. Meanwhile, P‐cluster maturation has not yet been investigated in the vanadium or iron‐only nitrogenases.

## 4 Conclusions

In the present study, we used a combination of biochemical techniques coupled to EPR spectroscopy and XAS to obtain a better understanding of the P‐cluster of nitrogenases, in particular in the VFe protein. Using well‐described affinity chromatography procedures, we were able to successfully isolate MoFe, apo‐MoFe, VFe, and apo‐VFe proteins from *A. vinelandii* cells. The analysis of the samples via SDS‐PAGE and NativePAGE revealed a heterogeneous composition of the VFe and the apo‐VFe samples with a lower content in α‐subunit, suggesting a mixture of complete hexamers and tetramers, respectively, with complexes lacking an α‐subunit. As the P‐cluster bridges the α‐ and β‐subunits, the presence of α_1_β_2_δ_2_ and α_1_β_2_ complexes implies the presence of incomplete P‐clusters in the protein samples. Our EPR studies suggest that VFe contains similar P‐clusters to MoFe, with spectroscopic differences arising from a fraction of immature P‐clusters. In contrast, we demonstrate that apo‐VFe does not contain any mature P‐cluster.

Additional EPR signals observed in the present work for VFe and apo‐VFe, both in perpendicular and parallel mode, could originate from immature or fragmented P‐clusters potentially with a [Fe_4_S_4_] clusters‐based structure, as has been proposed in previous studies.[^23^, ^24^, ^28^]

Apo‐forms of enzymes are often used to understand the cofactor, i. e. their spectra are often measured to provide a background that should be subtracted from holo‐protein measurements to provide a spectrum corresponding to the cofactor only. This has been used for MoFe, for which it has been shown that the P‐cluster has similar properties in the holo‐ and the apo‐form, although conformational changes do occur in the protein during cofactor insertion. Our work shows that such a subtraction process cannot be used in studies on VFe and its cofactor.

To conclude, the present study represents an essential step in our understanding of the differences between MoFe and VFe P‐clusters, and in particular the variations to the apo‐forms of these proteins.

## Conflict of Interests

The authors declare no conflict of interest.

5

## Supporting information

As a service to our authors and readers, this journal provides supporting information supplied by the authors. Such materials are peer reviewed and may be re‐organized for online delivery, but are not copy‐edited or typeset. Technical support issues arising from supporting information (other than missing files) should be addressed to the authors.

Supporting Information

## Data Availability

All other relevant data generated and analyzed during this study, which include experimental, spectroscopic, and computational data, are available in the Edmond Open Research Data Repository at https://doi.org/10.17617/3.D7EBHS and in the Supporting Information.
