# Supplementary material for: Understanding the P‐Cluster of Vanadium Nitrogenase: an EPR and XAS Study of the Holo vs. Apo Forms of the Enzyme
Source: Chembiochem. 2024 Dec 2;26(3):e202400833. doi: 10.1002/cbic.202400833 (PMC11823357; doi:10.1002/cbic.202400833)
Supplement: Supplementary file 1 — Supporting Information [file CBIC-26-e202400833-s001.pdf]

ChemBioChem

Supporting Information

Understanding the P-Cluster of Vanadium Nitrogenase: an EPR and XAS Study of the Holo vs. Apo Forms of the Enzyme

Isis M. Wahl, Kushal Sengupta, Maurice van Gastel, Laure Decamps,* and Serena DeBeer*

Supporting Information

for

Understanding the P-cluster of Vanadium Nitrogenase: an EPR and XAS study of the holo *vs.* apo forms of the enzyme

Isis M. Wahl,[a] Kushal Sengupta,[a] Maurice van Gastel,[b] Laure Decamps,[a,*] Serena DeBeer[a,*]

[a]Department of Inorganic Spectroscopy, Max Planck Institute for Chemical Energy Conversion, Mülheim an der Ruhr, 45470, Germany

[b]Department of Molecular Theory and Spectroscopy, Max-Planck-Institut für Kohlenforschung, Kaiser-Wilhem-Platz 1, Mülheim an der Ruhr, 45470, Germany

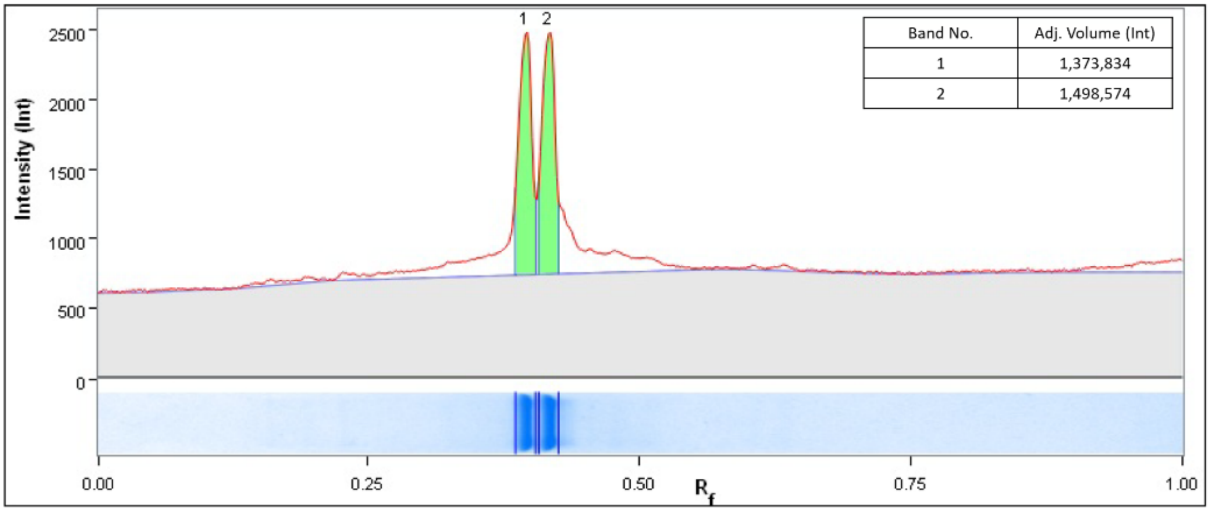

Figure S1. Densitometry analysis of the SDS-PAGE of MoFe using a Novex™ 4-20%, Tris-Glycine system. Image and analysis made using BioRad Image Lab software.

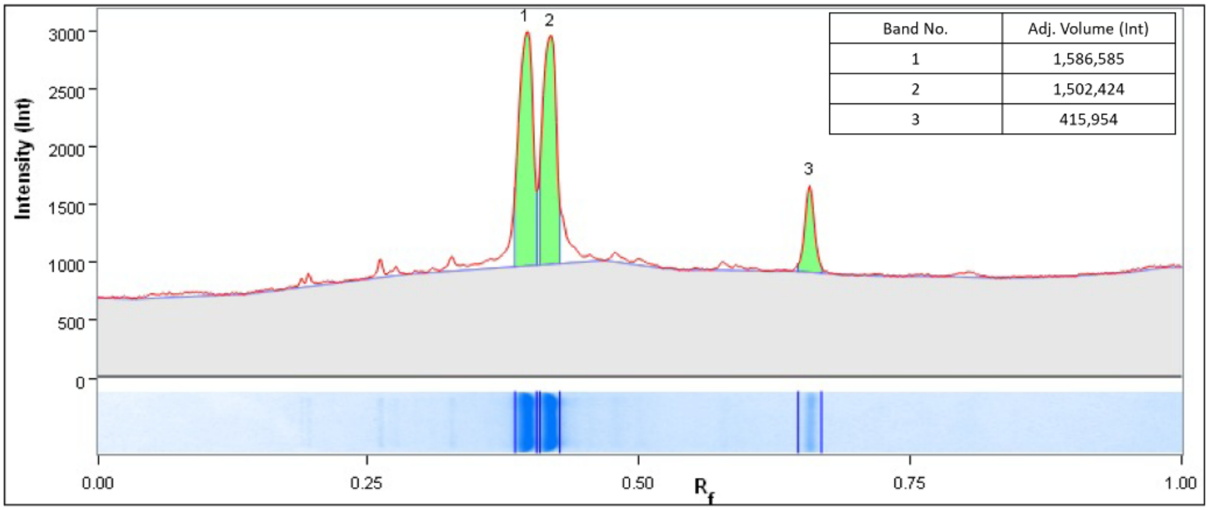

Figure S2. Densitometry analysis of the SDS-PAGE of apo-MoFe using a Novex™ 4-20%, Tris-Glycine system. Image and analysis made using BioRad Image Lab software.

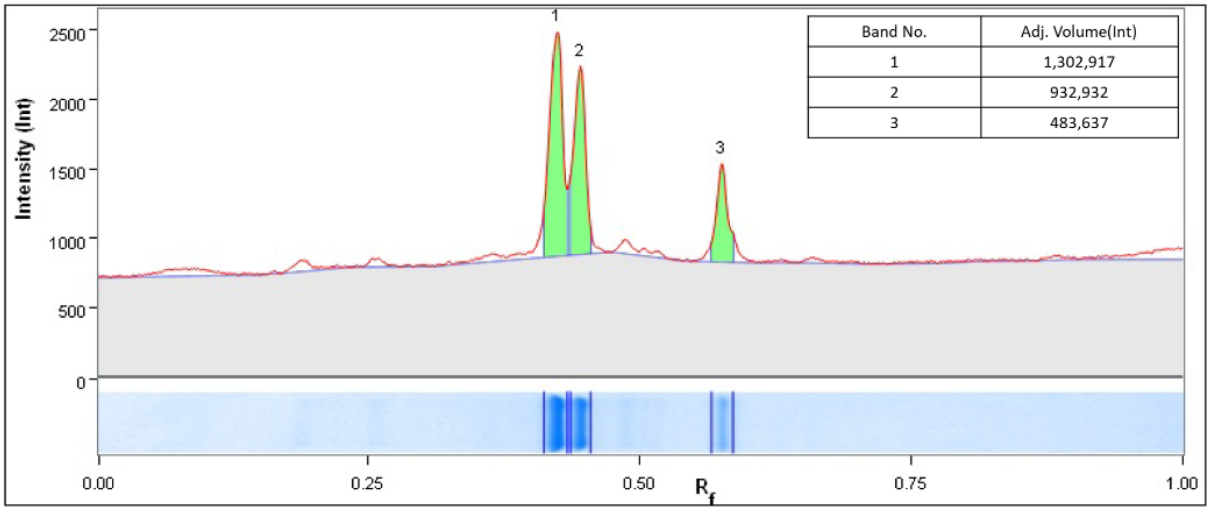

Figure S3. Densitometry analysis of the SDS-PAGE of VFe using a Novex™ 4-20%, Tris-Glycine system. Image and analysis made using BioRad Image Lab software.

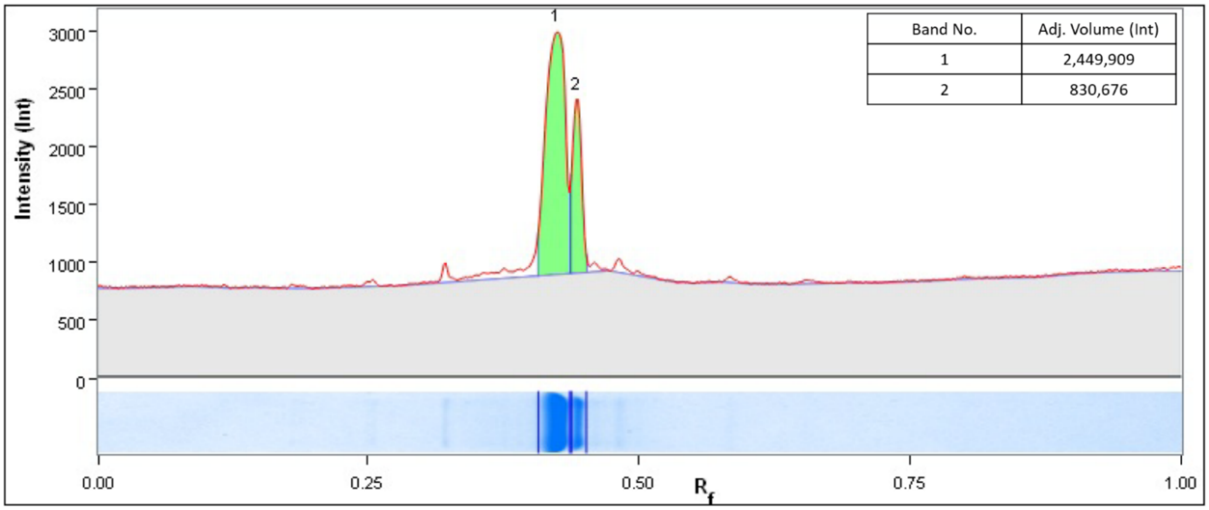

Figure S4. Densitometry analysis of the SDS-PAGE of apo-VFe using a Novex™ 4-20%, Tris-Glycine system. Image and analysis made using BioRad Image Lab software.

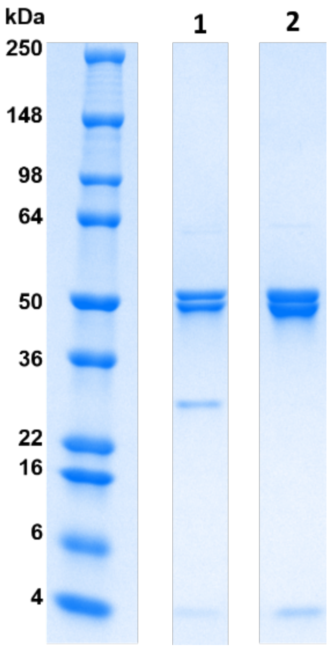

Figure S5. SDS-PAGE Novex™ 4-20%, Tris-Glycine of VFe isolated after a size-exclusion purification following isolation via Strep-Tag affinity purification. Lane 1 represents an early fraction containing the VnfJ protein and Lane 2 a later fraction lacking VnfJ.

Table S1. Summary of EPR signals observed in in this work and their tentative assignment.

	**EPR signals perpendicular mode**						**EPR signals parallel mode**		
	**DT-reduced**	**Spin System**	**Assignment**	**IDS-oxidized**	**Spin System**	**Assignment**	**IDS-oxidized**	**Spin System**	**Assignment**
**VFe**	$g = 6.69$	$S = 5/2$	possibly FeVco	$g = 6.69$	$S = 5/2$	possibly FeVco			
	$g = 5.48, 4.37, 3.97$	$S = 3/2$	possibly FeVco	$g = 5.48, 4.37, 3.97$	$S = 3/2$	possibly FeVco	$g = 12$	$S = 3$ or 4	P^{2+}
	$g = 2.05, 1.93, 1.90$	$S = 1/2$	P-cluster fragment or immature P-cluster	$g = 2.04, 2.00$	?	P-cluster fragment or immature P-cluster			
**apo-VFe**	$g = 2.05, 1.93, 1.90$	$S = 1/2$	P-cluster fragment or immature P-cluster	$g = 4.3$<br>$g = 2.09, 2.01$	?<br>?	P-cluster fragment or immature P-cluster	-	-	-
**MoFe**[1]	$g = 4.34, 3.66, 2.01$	$S = 3/2$	FeMoco	$g = 4.34, 3.66, 2.01$	$S = 3/2$	FeMoco	$g = 12$	$S = 3$ or 4	P^{2+}
**apo-MoFe**	-	-	-	-	-	-	$g = 12$	$S = 3$ or 4	P^{2+}

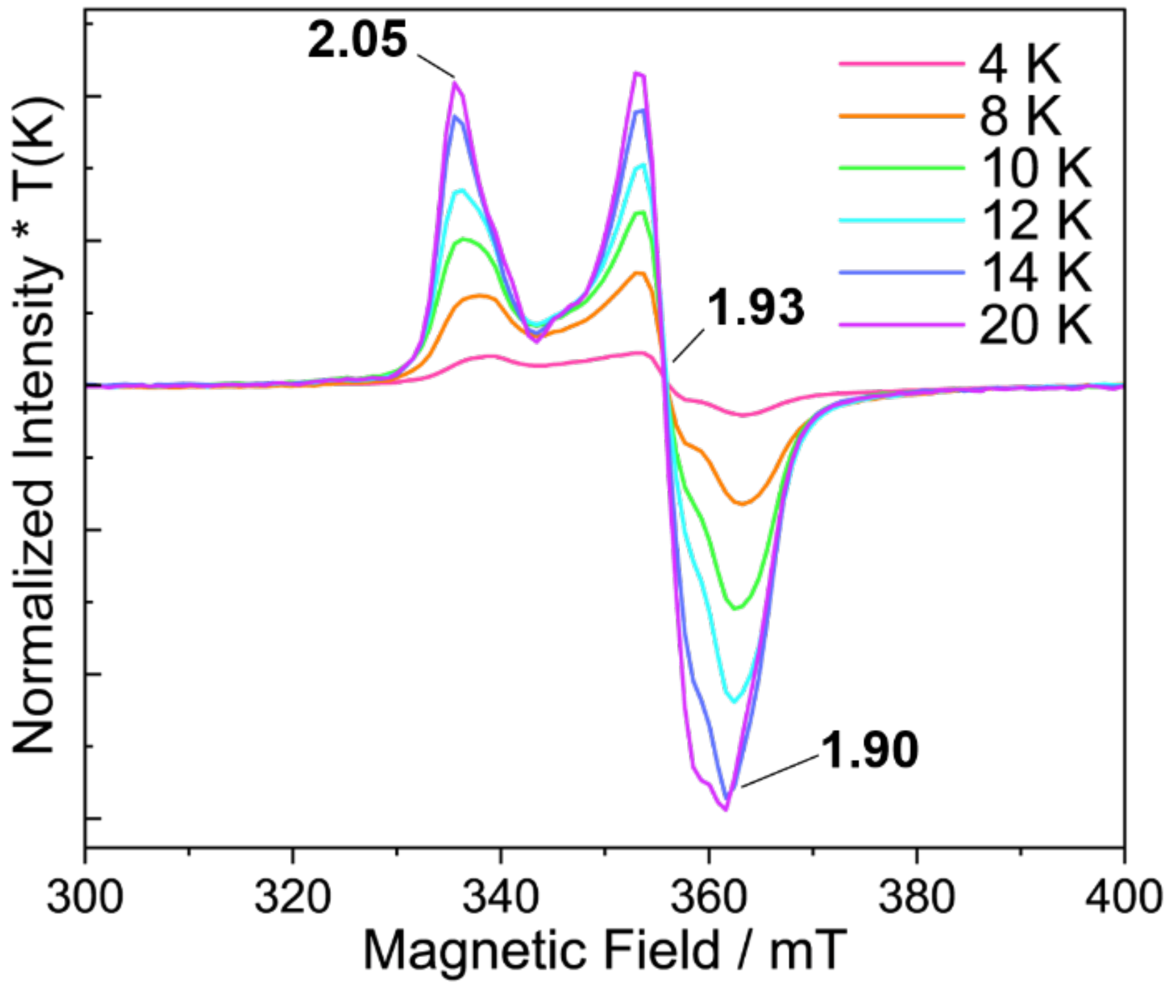

Figure S6. X-band perpendicular-mode EPR spectra of the temperature dependence experiment of apo-VFe protein (140 μM). EPR conditions: temperature as indicated in the graph; microwave frequency, 9.63 GHz; microwave power, 2 mW; modulation amplitude, 7.46 G; Each trace is the average of 5 scans.

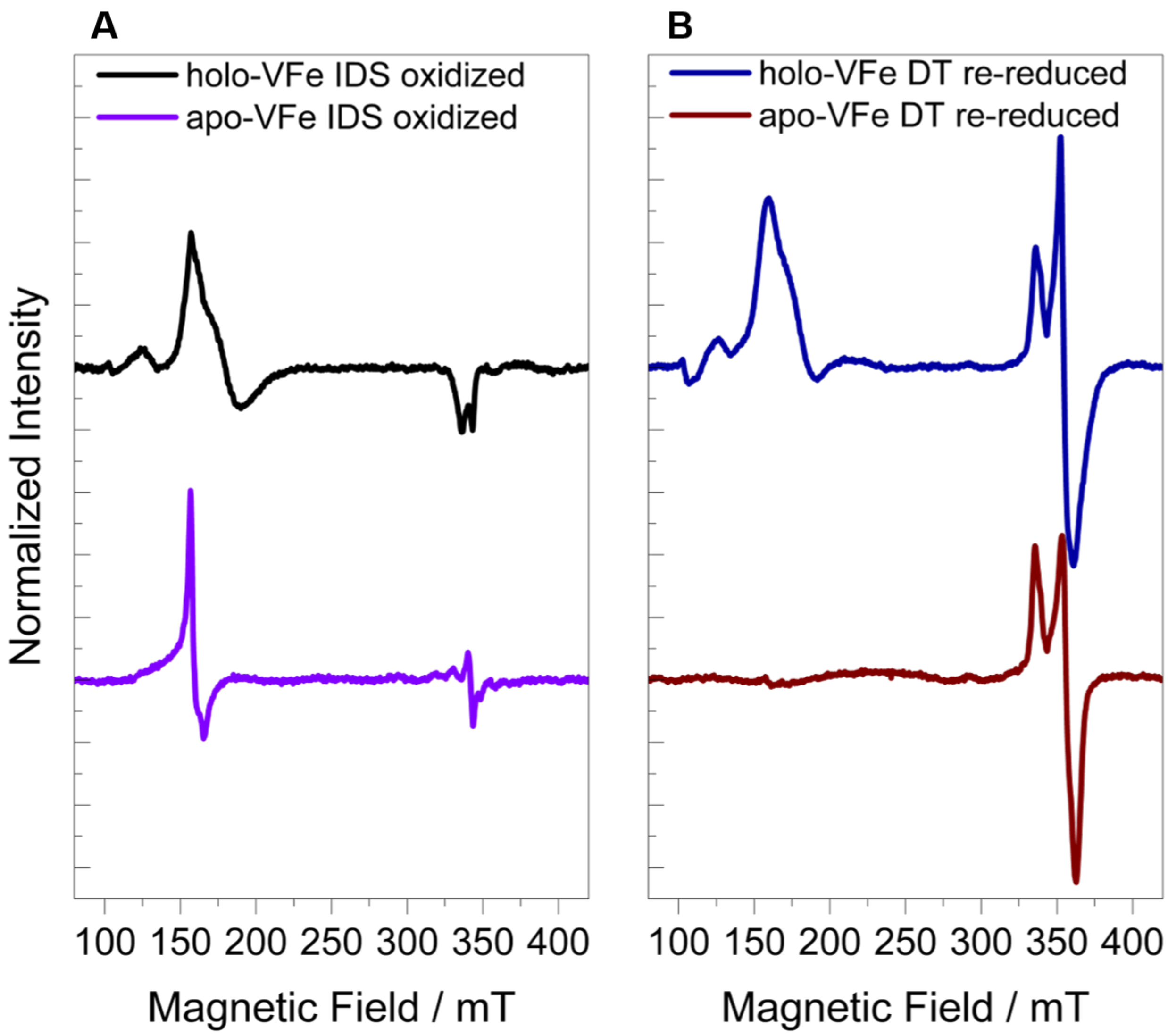

Figure S7. (A) X-band perpendicular mode EPR of IDS oxidized VFe (black) and apo-VFe (purple). Both samples were at a concentration of 140 μM and oxidized by 10x excess of IDS. (B) X-band perpendicular mode EPR of re-reduced VFe (blue) and apo-VFe (red). These samples were prepared by removing the excess of IDS present on the samples in (A) using a NAP-5 column, re-concentrating the samples to ~140 μM using a 100 kDa MWCO centrifugal filter unit (Millipore) and then using reducing the samples with 5 mM DT. EPR conditions: temperature14 K; microwave frequency 9.63 GHz; microwave power 5 mW; modulation amplitude 7.46 G. Each trace is an average of 10 scans.

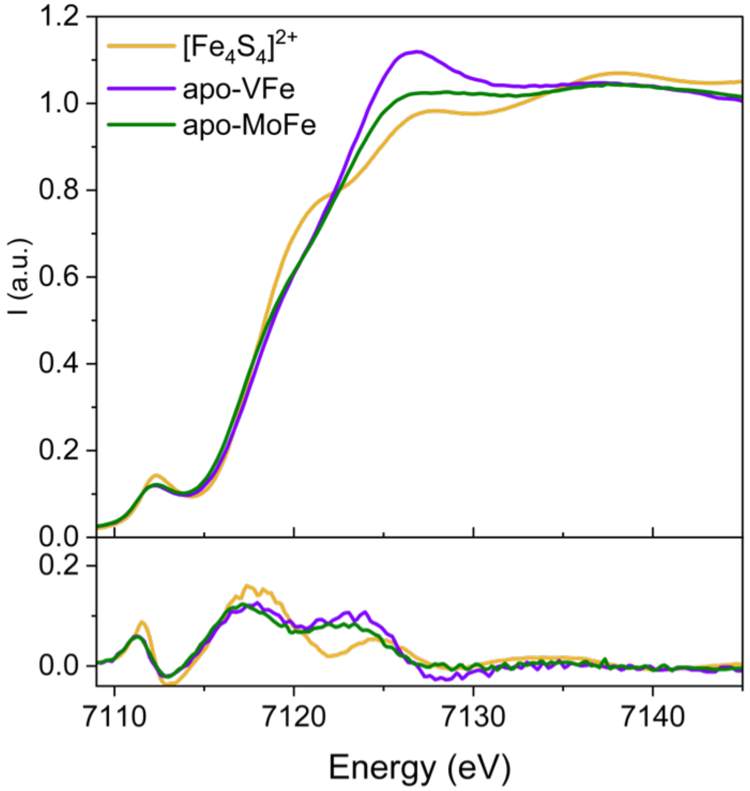

Figure S8. X-ray absorption spectra of $(NBu_4)_2[Fe_4S_4(SPh)_4]$ (yellow), apo-VFe (purple), apo-MoFe (green). Normalized PFY-detected Fe K-edge XAS of frozen samples (top) and corresponding first derivatives (bottom).

References

1. Van Stappen, C., Decamps, L. & DeBeer, S. Preparation and spectroscopic characterization of lyophilized Mo nitrogenase. *Journal of Biological Inorganic Chemistry* **26**, 81–91 (2021).
